# Production of Oncolytic Measles Virus in Vero Cells: Impact of Culture Medium and Multiplicity of Infection

**DOI:** 10.3390/v16111740

**Published:** 2024-11-06

**Authors:** Dustin Eckhardt, Jana Mueller, Jonas Friedrich, Jan-P. Klee, Irakli Sardlishvili, Lars E. Walter, Stefanie Fey, Peter Czermak, Denise Salzig

**Affiliations:** 1 Institute of Bioprocess Engineering and Pharmaceutical Technology, University of Applied Sciences Mittelhessen, Wiesenstraße 14, 35390 Giessen, Germany; dustin.eckhardt@biotecmed.thm.de (D.E.); peter.czermak@lse.thm.de (P.C.); 2 Faculty of Biology and Chemistry, University of Giessen, Heinrich-Buff-Ring 17, 35392 Giessen, Germany

**Keywords:** multiplicity of infection (MOI), media adaption, chemically defined medium (CDM), serum-free medium (SFM), viral vaccines, vectors, virus-like particles (VLPs), cell culture research, process development

## Abstract

Oncolytic measles virus (MeV) is a promising anti-cancer treatment. However, the production of high titers of infectious MeV (typically 10^7^–10^9^ TCID_50_ per dose) is challenging because the virus is unstable under typical production conditions. The objective of this study was to investigate how the multiplicity of infection (MOI) and different media—a serum-containing medium (SCM), a serum-free medium (SFM) and two chemically defined media (CDM)—affect MeV production. We infected Vero cells at MOIs of 0.02, 0.2 or 2 TCID_50_ cell^−1^ and the lowest MOI resulted in the largest number of infected cells towards the end of the production period. However, this did not equate to higher maximum MeV titers, which were similar for all the MOIs. The medium had a moderate effect, generating maximum titers of 0.89–2.17 × 10^6^, 1.08–1.25 × 10^6^ and 4.58–9.90 × 10^5^ TCID_50_ mL^−1^ for the SCM, SFM and CDM, respectively. Infection at a low MOI often required longer process times to reach maximum yields. On the other hand, a high MOI requires a large amount of MeV stock. We would therefore recommend a mid-range MOI of 0.2 TCID_50_ cell^−1^ for MeV production. Our findings show that SCM, SFM and CDM are equally suitable for MeV production in terms of yield and process time. This will allow MeV production in serum-free conditions, addressing the safety risks and ethical concerns associated with the use of serum.

## 1. Introduction

Cancer is the second leading cause of death worldwide [1]. An emerging cancer treatment approach is the use of oncolytic viruses, such as the Measles morbillivirus (MeV) vaccine strains [2], which are being tested in clinics for the treatment of ovarian cancer and glioma [3]. In addition to its ability to infect and lyse tumor cells [4], the MeV also induces systemic anti-tumor immune responses [5]. The high affinity of the MeV for tumors reflects the uptake of the virus via surface receptors such as Nectin-4, CD150/SLAM and the ubiquitous receptor CD46, which are commonly overexpressed in cancer cells [6,7,8]. Infected cells change morphologically as they fuse with adjacent cells, form multi-nuclear giant cells (syncytia) and finally lyse. This virus-mediated cell damage, also known as the cytopathic effect, is one of the mechanisms of action of oncolytic virotherapy [9].

A high dose of infectious MeV is necessary for a successful treatment: typically 10^7^–10^9^ TCID_50_ [10], and in one exceptional case a systemic application of 10^11^ TCID_50_ [11]. This requires highly efficient production processes, whereby the MeV is produced under optimal conditions with the highest possible yield. Potent host cells (e.g., Vero or MRC-5 cells) enable the production of high titers of the MeV up to a bioreactor scale [12,13,14,15], but a major challenge is the physicochemical instability of the virus. The MeV is rapidly inactivated at the optimum temperature for the cultivation of mammalian cells (37 °C) [16,17] and is also highly unstable at a pH of <7, which is problematic for MeV production in systems without pH regulation [16,17]. It is therefore necessary to determine the optimal time of harvest (TOH) with precision, because missing this optimal time can result in MeV losses of several log units within hours [18]. In addition to the TOH, the multiplicity of infection (MOI) is a critical process parameter that must be optimized to maximize virus yields. The MOI is defined as the ratio of infectious virus particles (or their relative units) to host cells at the time of infection (TOI). Theoretically, high MOIs allow the simultaneous infection of all host cells whereas low MOIs result in a proportion of cells being infected at the beginning of the process. The non-infected cells continue to proliferate and are then infected by newly produced viruses during secondary infections. This initially leads to populations of infected and non-infected cells, but it is likely that all cells are infected later in the process. A high MOI is beneficial for short process times but requires a larger amount of virus stock for inoculation. The low MOI strategy allows the growth of non-infected cells, which results in a greater number of cells being infected at the end [14]. Given that a MeV infection is a statistical process, it can be reasonably assumed that an increase in the number of virus particles will inevitably lead to an increase in the number of infected (and thus virus-producing) cells [18]. Previous studies have only considered the cell concentration at the time of infection without analyzing the number of cells actually infected [13,14]. Monitoring the number of infected cells would help a priori to determine the most appropriate MOI for an optimal space–time yield of infectious MeV. However, it must be noted that a successful infection with the MeV does not necessarily result in high virus titers. This is because the virus must replicate within the host cell and be released in order to achieve high yields [19].

A critical element for an effective MeV production process is the choice of the cell culture medium, which must be optimized for the infection process, virus replication and release. High MeV yields have already been achieved in a serum-containing medium (SCM) using Vero cells in a microcarrier-based stirred-tank reactor (STR) [14]. However, the use of serum in cell culture media has several disadvantages including high costs, ethical concerns, and (especially in immunocompromised cancer patients) contamination with pathogens and the risk of serum-induced allergic reactions. Furthermore, the higher protein load of serum supplemented media can lead to increased foaming in the STR processes, which in turn can result in cell discharge and reduced oxygen transfer from the headspace. In addition, the undefined nature of serum leads to batch-to-batch variations, which directly affect the MeV quality and raise safety concerns. The use of a serum-free medium (SFM) and a chemically defined medium (CDM) are therefore preferred in the biopharmaceutical industry [20]. A SFM was previously tested for MeV production and showed promising results in terms of yield and process efficiency [13,15]. However, little information is available about the production of the MeV in CDM.

Here, we investigated the effect of the MOI and different media on MeV production. We adapted Vero cells for growth in one SFM and two CDMs to determine the suitability of these media for MeV production in static cultivation systems. The media were compared to a standard Dulbecco’s modified Eagle’s medium (DMEM) supplemented with 10% fetal bovine serum (FBS). To evaluate the influence of the MOI on static MeV production, we used a model MeV strain (Section 2.3), allowing the ratio changes between total cells and MeV-infected cells to be monitored by flow cytometry. In addition, we used fluorescence microscopy to investigate the course of the infection and its spread during static cultivation, and determined the concentrations of the relevant substrates and metabolites in the culture supernatant.

## 2. Materials and Methods

### 2.1. Cultivation of Vero Cells

The adherent cell line Vero-B4 was purchased from DSMZ (#ACC 33). For general maintenance, the cells were seeded into T-75 or T-175 tissue culture flasks with venting caps (Sarstedt, Nümbrecht, Germany; #83.3911.002 or #83.3912.002) at 5–10 × 10^3^ cells cm^−2^ with 0.28 mL cm^−2^ culture medium and were incubated at 37 °C in an 8% CO_2_ atmosphere. The cells were passaged after reaching 90–100% confluence.

For harvesting, the cell layer was washed twice with phosphate-buffered saline (PBS; without Ca^2+^/Mg^2+^; Sigma-Aldrich, St. Louis, MI, USA; #D8537) and covered with at least 0.08 mL cm^−2^ trypsin/EDTA (prepared from a 10× stock in PBS; Biochrom, Berlin, Germany; #L2153) or a 1× TrypZean solution (recombinant trypsin without components of animal origin; Sigma-Aldrich; #T3449). Trypsinization was carried out at 37 °C in an 8% CO_2_ atmosphere until the cells detached from the surface.

The enzymatic reaction was stopped by adding three times the trypsin volume of the SCM or, in the case of SFM/CDM, by adding a trypsin inhibitor (stock solution 1 mg mL^−1^ in PBS; Sigma-Aldrich, #T6522) using the same volume as TrypZean. The detached cells were centrifuged (250× *g*, 5 min, room temperature) and resuspended in a fresh medium before seeding into new T-flasks or use for MeV production. When cultured in an SCM, the centrifugation step was omitted and the cell suspension was used directly. Cell concentrations were determined using a Scepter 2.0 cell counter (Merck, Darmstadt, Germany; #PHCC20060) and the corresponding Scepter 60 µm sensors (Merck, #PHCC60500). The cell expansion factor was calculated by dividing the cell density at the time of harvest by the seeding cell density.

### 2.2. Adaption of Vero Cells to SFM and CDM

Vero cells were adapted from a standard DMEM (Gibco Life Technologies, Carlsbad, CA, USA; #41965062), supplemented with 10% FBS (Capricorn Scientific, Ebsdorfergrund, Germany; #FBS-11A), to one SFM (VP-SFM; Gibco Life Technologies, #11681020), supplemented with 4 mM l-glutamine (200 mM stock; Sigma-Aldrich, #G7513), and two non-commercial research CDMs (73614C and 73835C) kindly provided by Merck (Germany) and supplemented according to their instructions.

After thawing the Vero cells in standard medium (DMEM with 10% FBS) and the first passaging, the cells were adapted to the new media during the next passage by successive media exchanges over 3 days. We started with a 75/25 ratio of standard/new medium from 0.33 days post seeding (dps), then switched to 37.5/62.5 from 1 dps, then to 7.5/92.5 from 2 dps, and finally to 100% VP-SFM or CDM from 3 dps. The cells were then cultured and expanded for three further passages before master cell banks (MCBs) were generated. For this purpose, we used a chemically defined freezing medium without components of animal origin (10 mg mL^−1^ human serum albumin; Sigma-Aldrich, #A1653-5G), 9 mg mL^−1^ NaCl, 10% (*v*/*v*) dimethyl sulfoxide (Sigma-Aldrich, #D2650) in demineralized water). Working cell banks (WCBs) were generated after thawing a MCB vial and three subsequent passages.

### 2.3. Measles Virus Strain

The infectious model strain MeV NSe ld-EGFP (derived from the Edmonston B-derived MeV vaccine strain NSe) was kindly provided by Prof. Guy Ungerechts, from the National Center for Tumor Diseases (Heidelberg, Germany). The virus genome carries an *eGFP* transgene, and therefore an enhanced GFP is expressed by the host cells after an infection.

### 2.4. MeV Production in Six-Well Plates

Vero cells (from the WCB) were seeded at 10,000 cells cm^−2^ in six-well tissue culture plates (Sarstedt, #83.3920) with 0.28 mL cm^−2^ of the appropriate medium and were incubated at 37 °C in an 8% CO_2_ atmosphere. The cells were infected 0.83 dps at the MOIs of 0.02, 0.2 and 2 TCID_50_ cell^−1^ (±0.5 log_10_ deviation possible for all the MOIs caused by the limit of the TCID_50_ assay [21,22,23]) by adding the required MeV stock (MeV in the appropriate media, stored at −80 °C) volume. We assumed that the changes in cell number from seeding to the TOI were negligible (in relation to the MOI with log_10_-level differences) due to the lag phase, which is ~1 d. The infectious virus titers of the MeV stocks were determined using a TCID_50_ assay in eight technical replicates. Mock-infected Vero cell cultures were used as controls. The MeV-infected cultures in the standard medium DMEM/FBS served as reference cultures. To increase the stability of the virus [16,17], the temperature was reduced to 32 °C immediately after the infection, as previously described [14]. Because the volume of the MeV stock we added was dependent on the culture medium and MOI, the medium was exchanged in all cultures at 1.25 days post-infection (dpi) to ensure the same media conditions and filling volumes. Based on our experience, the number of viruses released at this time point is negligible.

For each medium, culture supernatant samples were taken from one well per day for further analysis. The TCID_50_ assay samples were stored at −80 °C and cells were detached with trypsin for an immediate analysis by flow cytometry. The cell densities of the total and infected (GFP^+^) cells were recalculated from the cell concentration results. As the resuspension of the trypsinized MeV-infected cells had to be comparatively rough, especially in the later course of infection (due to sticky cell aggregates), it is likely that a certain degree of mechanical cell lysis was anticipated. This intrinsic error during cell quantification should be considered regarding the total cell densities. In addition, one well containing cells growing in each medium was prepared for fluorescence microscopy at 3 and 5 dpi.

### 2.5. Flow Cytometry

Harvested cells were, if necessary, diluted to a concentration of 1–5 × 10^5^ cells mL^−1^. We transferred 200 μL of the cell suspension to a 96-well plate with a flat bottom for an analysis using a Guava easyCyte flow cytometer (Cytek Biosciences, Fremont, CA, USA) and guavaSoft Incyte software (version 4.5). The cell population was separated from debris by creating a gate in the sideward scatter (SSH-HLin)–forward scatter (FSC-HLin) dot plot. In the selected gate, 1300–5000 events were recorded per sample at an average flow rate of 0.59 μL s^−1^. The wells were mixed for 6 s between individual measurements. The fluorescence signal was detected after passing a 583/26 nm bandpass filter with the photomultiplier as *Yel-B-H log* and logarithmically amplified (five decades). The fluorescence signal of the gated cell population was exported as an abundance histogram (counts vs. Yel-B-H log). The autofluorescence of the non-infected cells was amplified to be close to the ordinate. The fluorescence intensity threshold for the GFP^+^ (infected) cells was set by filtering out >98% of the non-infected control cells. Cells with a fluorescence signal exceeding the measurement range were registered as the maximum signal value. The experiments were carried out in technical duplicates (data points represent the mean value).

### 2.6. Fluorescence Microscopy

The infection status of the adherent Vero cell cultures was also checked by fluorescence microscopy at 3 and 5 dpi. The first day on which a sufficient GFP signal was detectable by fluorescence microscopy was 3 dpi, whereas 5 dpi was the last day on which intact cells could still be imaged in the cultures with a high MOI. The GFP signal was used to determine the number of MeV NSe ld-EGFP-infected cells, whereas the cell nuclei were counterstained with 4′,6-diamidino-2-phenylindole (DAPI; AppliChem GmbH, Darmstadt, Germany; #A1001,0010) to determine the total number of cells. Vero cell cultures were washed twice with 0.1 mL cm^−2^ PBS and then mixed with 0.1 mL cm^−2^ DAPI staining solution (0.1 µg mL^−1^ DAPI in 5% (*v*/*v*) methanol in PBS) and incubated for 30–40 min at room temperature in the dark. The cells were not fixed beforehand in order to preserve their native state. After washing with PBS, fluorescence images were captured automatically using the Cytation 3 (Agilent Technologies (BioTek), Santa Clara, CA, USA) and associated Gen 5 software (Agilent BioTek). We used 2.5× or 10× objectives (GFP and DAPI). The overview images were each stitched from four images (2 × 2) using the 2.5× objective.

### 2.7. Determination of the Infectious MeV Titer

The virus titer was determined using a TCID_50_ (median tissue culture infectious dose) assay. A Vero cell suspension with 50,000 cells mL^−1^ in DMEM (10% FBS) was prepared and 0.2 mL of the cell suspension was pipetted into all wells of a 96-well tissue culture plates (Sarstedt, #83.3924). The plates were incubated at 37 °C in an 8% CO_2_ atmosphere for at least 4 h. The MeV samples were serially diluted in rows of separate 96-well plates in 1:10 dilution steps (10^0^ to 10^−11^) with DMEM (10% FBS). The pipette tips were changed at each dilution step. We transferred 30 μL of each sample dilution to eight wells containing Vero cells and incubated the plates for 6–7 days at 32 °C in an 8% CO_2_ atmosphere. After incubation, we used the Cytation 3 and Gen 5 software to capture automatic images of GFP expression with a 2.5× objective (six per well (2 × 3), stitched) to confirm an infection with the MeV NSe ld-EGFP. The TCID_50_ titer was determined based on Spearman–Karber calculations of the 50% endpoint dilution [24,25].

### 2.8. Substrate and Metabolite Concentrations

Samples from the cultures were centrifuged (2000× *g*, 2 min, room temperature) and the glucose and lactate concentrations in the supernatant were determined using the Biosen C-line analyzer (EKF Diagnostics, Barleben, Germany). Ammonia, glutamine, glutamate and pyruvate were measured semi-automatically using the Cedex Bio Analyzer (Roche, Basel, Switzerland). Lactate dehydrogenase (LDH) enzyme activity in the supernatant was monitored as a marker of cell lysis using the same device. All the measurements were carried out according to the manufacturers’ instructions.

## 3. Results and Discussion

### 3.1. Growth Kinetics of Vero Cells Adapted to SFM and CDM

**Figure 1:**
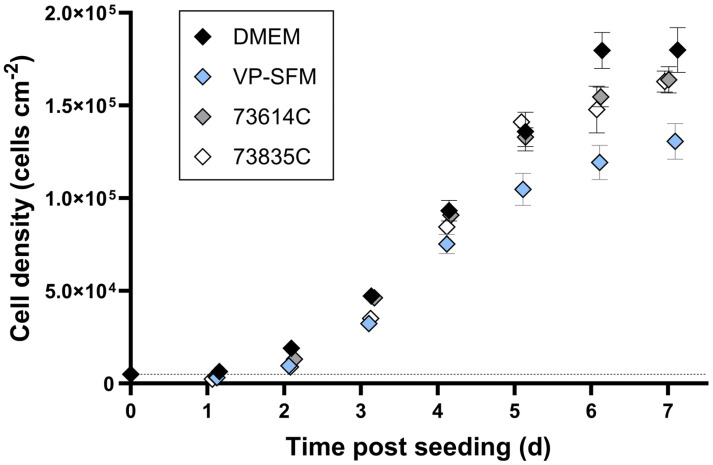
Growth kinetics of adapted Vero cells in static batch cultivation. Vero cells were seeded in six-well plates (5000 cells cm^−2^_,_ marked by dashed line) and incubated at 37 °C in an 8% CO_2_ atmosphere. Cells from three wells per medium were detached using trypsin every day and counted using a Scepter 2.0 cell counter (n = 3, mean ± SD).

We compared the growth kinetics of the Vero cells adapted to an SFM and two CDMs to the cells in standard DMEM during static cultivation (Figure 1).

The highest cell densities ~6 days after seeding were achieved in the DMEM (10% FBS) with 180,000 ± 10,000 cells cm^−2^ (=1.60 ± 0.09 × 10^6^ total cells). The two CDMs (73614C and 73835C) and the VP-SFM resulted in cell densities of 120,000–155,000 cells cm^−2^ (=1.06–1.37 × 10^6^ total cells). The CDMs and SFM therefore reached sufficient cell densities, with growth kinetics largely comparable to an SCM. A direct comparison indicated the slightly weaker overall performance of the VP-SFM.

To our knowledge, there are only a few growth kinetics of Vero cells in static cultivation systems. In one of them, maximum Vero cell densities of ~280,000 cells cm^−2^ in a SFM and ~380,000 cells cm^−2^ in a SCM could be achieved within 6.25 days [26]. However, the seeding densities in this study were 80,000 cells cm^−2^, which were already 16-fold of the ones in our experiments. In another study, maximum cell densities of 236,000 cells cm^−2^ were achieved in a SFM within 7 days of static cultivation starting with a seeding density of 20,000 cells cm^−2^ [27]. To better compare our results with those of the other studies, we calculated the cell expansion factor. Here, we reached with 36 or 24–31 much higher expansion factors than [26] with 3.5 or 4.8 and [27] with 11.8.

### 3.2. Influence of MOI on Virus Yields and TOH

**Figure 2:**
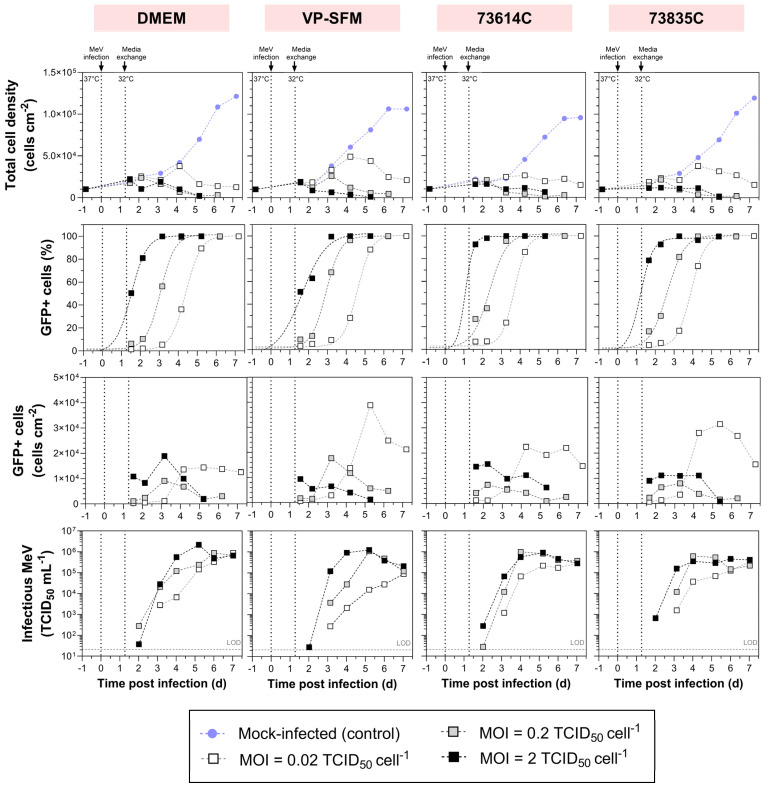
Measles virus production in adherent Vero cells at different MOIs in six-well plates. After the infection of Vero cells with MeV, the cultivation temperature was reduced from 37 to 32 °C and a complete media exchange was carried out ~1 day later. The concentration of total cells and the ratio of infected cells (%GFP^+^ cells) in four media were determined by flow cytometry, allowing for the recalculation of cell densities. Criterion for GFP^+^: fluorescence intensity greater than the mock-infected control. Sigmoidal curve fitting of %GFP^+^ cells over time, forced through points [–0.82;0] and [0;0] (time points of cell seeding and MeV infection). MeV titers were determined using the cell-based 50% endpoint dilution method (TCID_50_ assay). LOD: limit of detection.

We compared the influence of the MOI (0.02, 0.2 and 2 TCID_50_ cell^−1^) on static MeV production in the Vero cells adapted to a SFM and a CDM and those in a SCM (Figure 2). We analyzed the course of MeV production by the number of infected cells (GFP^+^ cells) per growth area and the infectious MeV titer in the culture supernatant.

#### 3.2.1. MOI-Dependent Growth Kinetics

We observed MOI-dependent cell growth in all the media during MeV production. The cultures infected at an MOI of 2 or 0.2 experienced a short cell growth phase and reached a maximum cell density of 12,000–24,000 cells cm^−2^ at 1.5–2.2 dpi. The fact that high MOIs limits cell growth has already been shown in dynamic MeV productions with Vero cells in regulated STRs [14]. Our results confirmed this correlation for static, unregulated cultivation systems—regardless of the medium used.

The only exception was observed in the VP-SFM at an MOI of 0.2, where the maximum cell density was reached at a later point in time (26,010 cells cm^−2^ at 3.2 dpi). After reaching the maximum, the cytopathic effect of the MeV reduced the total cell number, so that fewer than 6400 cells cm^−2^ were left at 5–6 dpi. This probably reflected a combination of cell lysis and the fusion of the infected cells with adjacent cells, leading to the formation of polynucleated giant cells (syncytia) [9]. The detachment of apoptotic cells from the growth surface may also have contributed because only the adherent cells were quantified after washing and detaching the cell layer. Moreover, the virus-induced alterations in cell metabolism and cell cycle progression also limit cell proliferation.

In contrast, the lowest MOI (0.02) prolonged cell proliferation up to 4.2 ± 0.047 dpi, regardless of the culture medium. The peak cell density was therefore up to 0.74 times higher in DMEM/73614C, and even up to 2.31 times higher in VP-SFM/73835C, compared to the MOIs of 0.2 and 2. Even at 7 dpi, more than 12,570 cells cm^−2^ remained in the cultures infected at an MOI of 0.02.

**Figure 3:**
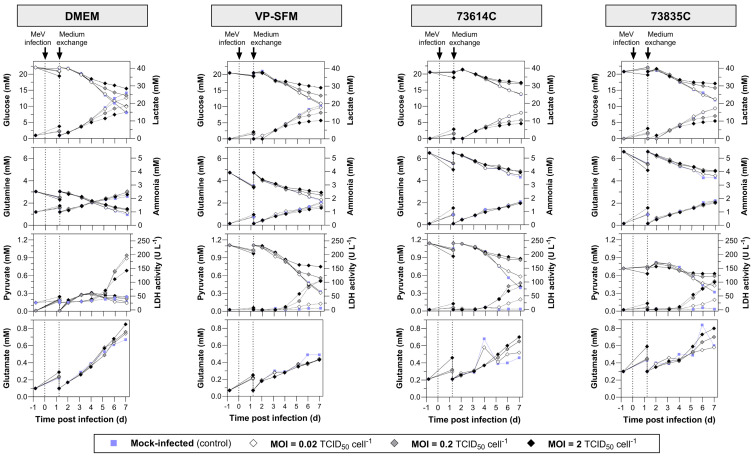
Substrate and metabolite concentrations and LDH activity during the static production of MeV in DMEM (10% FBS), VP-SFM (4 mM Gln), CDM 73614C and CDM 73835C (both according to Merck’s instructions). Following the infection of Vero cells with MeV, the cultivation temperature was reduced from 37 to 32 °C and a complete media exchange was carried out ~1 day later. Data points after medium exchange were not measured, but were copied from the origin data (–0.82 dpi). Solid lines (data plotted left): glucose, glutamine, pyruvate, glutamate. Dashed lines (data plotted right): lactate, ammonia, LDH activity.

The substrate uptake, lactate and ammonia accumulation, and LDH release are summarized in Figure 3. During MeV amplification, the glucose and glutamine concentrations remained above the critical values (glucose > 5 mM, glutamine > 0.5 mM), which were already defined for Vero cell STR cultivations with bolus feeds [28]. Ammonia accumulated to a maximum of 2.5 mM, which is only half the IC_50_ (50% reduction in growth) of 5 mM for Vero cells [29]. Lactate, which indicates exponential growth in uninfected Vero cell cultures, did not exceed the critical concentration of 19 mM in any of the cultures infected at MOIs of 0.2 or 2. At an MOI of 0.02, the lactate concentration 7 dpi was 14.6–23.2 mM and thus above or near to the critical value. The LDH activity in the culture supernatant increased in all the MeV-infected cultures (from 5 dpi at the latest), which can be explained by the lysis of the cells during the course of the MeV infection. Pyruvate (a component of VP-SFM and the CDMs) was consumed, remaining at the highest levels in the mock-infected cultures and at the lowest levels in the heavily MeV-infected (MOI 2) cultures. However, the Vero cells were not dependent on pyruvate given their ability to grow in DMEM, which lacks this compound. The slow and MOI-dependent consumption of substrates and the formation of metabolites in the MeV-infected cultures are associated with lower cell densities compared to the mock-infected cultures.

#### 3.2.2. MOI-Dependent Infection Kinetics

Infected cells were detected by flow cytometry based on the presence of GFP, providing evidence of successful MeV propagation at each tested MOI (Figure 2). The infection ratio increased over time and ultimately exceeded 95%. The higher the MOI, the less time required to reach 50% infected cells (1.36 ± 0.23 dpi at a MOI = 2, 2.75 ± 0.27 dpi at a MOI = 0.2 and 4.14 ± 0.36 dpi at a MOI = 0.02) and subsequently ~100% infection. This relationship can be explained by the variable number of completed infection cycles and the subsequent secondary infections.

Compared to an MOI of 2, it is evident that the lower MOIs lead to a multi-stage infection process in which newly formed viruses infect the remaining uninfected host cells. The longer expansion phase of Vero cells observed when using an MOI of 0.02 may therefore reflect the delayed spread of the infection through the cell population. As expected, the highest MOI resulted in a greater number of infected cells at the beginning of the process. Although the density of infected Vero cells increased somewhat later when using the medium MOI, the trends were similar, mirroring the observations regarding total cell density. The medium and high MOIs resulted in a maximum density of 7500–19,000 infected cells cm^−2^ 3 dpi at the latest. By 5 dpi, fewer than 6000 infected cells cm^−2^ were left. At the low MOI, we observed a sharp increase in the number of infected cells between 3 and 5 dpi, which then stayed more or less stable for 2–3 days in the DMEM and CDMs but decreased immediately in the VP-SFM. Importantly, the maximum number of infected cells was up to 4.16 times higher compared to the MOIs of 0.2 and 2 (with the exception of the DMEM, where the difference was minimal).

The hill slopes of the sigmoidal curves of the GFP^+^ cells over time (especially noticeable in the DMEM, with hill slopes of 1.11 ± 0.02) were mostly similar, indicating that the propagation of an infection is MOI-independent, and its course is only temporally shifted. Deviating hill slopes (e.g., in the VP-SFM with MOI = 2) probably reflected measurement inaccuracies combined with a low number of data points. For future studies, we would therefore recommend more measurements per day. Because the time intervals between “reaching 50% infected cultures”, depending on the MOI, were very similar in all the media, with Δt = 1.39 ± 0.16 (MOI 2 vs. 0.2) and Δt = 1.39 ± 0.11 (MOI 0.2 vs. 0.02), the replication cycle of the MeV NSe ld-EGFP appears to last ≤ 1.4 d.

Theoretically, an MOI of 2 TCID_50_ cell^−1^ should be sufficient for a simultaneous infection of the whole cell population. However, at the highest MOI, the proportion of infected cells at the beginning of the process (~1.6 dpi) was in some cases below 95%. Notably, the identification of the infected cells was delayed because a sufficient GFP accumulation is needed to distinguish the infected from the non-infected cells. Furthermore, the different cell cycle phases in the cell population and the uneven distribution of infectious virus particles on the host cells (Poisson distribution [30]) could explain the reduced infection efficiency at the TOI.

#### 3.2.3. MOI-Dependent MeV Yield

The influence of the MOI on the infection process and cell growth was reflected in the virus release profile. Consistent with the presence of more virus-infected cells at the beginning of the process with higher MOIs, the virus titer also increased more rapidly. The optimal TOH was therefore achieved 1 day sooner. On the other hand, there was little difference in the virus release profiles when comparing the medium and high MOIs in the CDMs (optimum TOH = 4–5 dpi). Even so, a change in the MOI from 2 to 0.2 did not substantially affect the maximum virus yield, which was 0.89–2.17 × 10^6^ TCID_50_ mL^−1^ in the DMEM (10% FBS), 1.08–1.25 × 10^6^ TCID_50_ mL^−1^ in the VP-SFM, and 4.58–9.90 × 10^5^ TCID_50_ mL^−1^ in the CDMs. These titers are similar to those reported for MeV produced in Vero cells in VP-SFM using unregulated systems [31]. It is notable that the previously reported results differed from our findings regarding an MOI-dependent TOH. Although lower MOIs were used, the maximum MeV titers were reached earlier. However, this discrepancy may reflect the use of a different MeV strain, suggesting a re-evaluation of the data is needed for each individual strain. In both cases, there is an MOI range that differs by one log level (MOIs 2 and 0.2 in our case, MOIs 0.1 and 0.01 previously [31]) but still shows a very similar MeV production profile over the process time. Presumably, this reflects the mainly small differences in the time course of the infected cells as well as host cell availability, as described above.

The subsequent loss of infectivity can be attributed to the virus inactivation rate, which is higher than its release rate. In addition to thermal inactivation, proteases released during cell lysis might damage the MeV proteins, such as the surface proteins required for adsorption (H protein and F protein). Furthermore, metabolites and debris released during cell lysis accumulate in the supernatant over time, probably affecting the structure and aggregation of the MeV, for example by changing the pH [16]. At MOIs of 0.2 and 2, the peak virus titer was accompanied by a drop in cell density (60–93% of X_max_). This suggests that a large quantity of virus particles is released by cell lysis. The titer increased almost continuously throughout the process with an MOI of 0.02. Given the limited timeframe, it remains unclear whether the virus titer would have increased had more time been available. The continued presence of infected cells in the cultures suggests that such an increase is likely. To achieve this, it would be necessary to ensure an adequate supply of nutrients and oxygen for MeV production and host cell survival, while maintaining optimal process conditions (e.g., pH and the concentrations of substrates and inhibitory metabolites).

The expectation that larger numbers of infected cells enhance the virus yield was not confirmed in our static batch cultivation experiment. This has also been shown in a host cell screening for MeV production, in which both the adherent and suspension cells were examined [19]. Although the MeV infection was successful and nearly complete in most of the experiments (six of nine host cell types, >95% were infected by the MeV), the maximum MeV titers varied by up to 4 log levels. The Vero cells proved to be the most productive host cells for the MeV [19]. However, the extent to which the proportion of MeV-infected Vero cells affects the maximum MeV yield was not investigated here. Even if more infected cells were available for MeV production at an MOI of 0.02, the maximum yield of infectious MeV was either similar to or even 0.5–1.2 log_10_ lower than an MOI of 0.2 or 2. One potential explanation is that we only assessed the GFP^+^ cells (compared to the mock-infected control). The different GFP intensities of the GFP^+^ cells, which were higher at higher MOIs, were not taken into account because this would have complicated the presentation of the results. Higher GFP intensities could represent a greater viral load per cell and thus lead to higher MeV production rates. However, given that GFP intensity can also be influenced by other transcriptional and translational factors, its relationship to MeV production is not clear.

Our findings align with those of previous studies that have demonstrated the positive effect of higher MOIs on MeV yields [12,32]. For the propagation of the MeV in MRC-5 cells, increasing the MOI from 0.001 to 0.01 increased the yield from 7.5 × 10^5^ to 7.5 × 10^6^ TCID_50_ mL^−1^ [12]. In contrast, other reports suggest that higher MeV yields are achieved by reducing the MOI, but at the expense of longer process times [32,33]. Moreover, the MeV production in Vero cells using a microcarrier-based STR process with various MOIs (0.0005, 0.001 and 0.02) resulted in MeV titers reaching almost the same order of magnitude [13].

The selection of an appropriate MOI involves a compromise between production time, virus yield and the required volume of virus stock [20] and also appears to be dependent on the MeV strain. Furthermore, any increase in cell lysis and the accumulation of inactivated MeV particles makes the medium more complex, which directly affects downstream processing. To generate a high yield of infectious MeV in a short process time while reducing the required MeV stock volume, among the three MOIs we tested, we would recommend an MOI of 0.2 for the production of Edmonston B-derived MeV strains in static cultivation systems.

#### 3.2.4. Additional Monitoring of the MeV Infection Course by Fluorescence Microscopy

**Figure 4:**
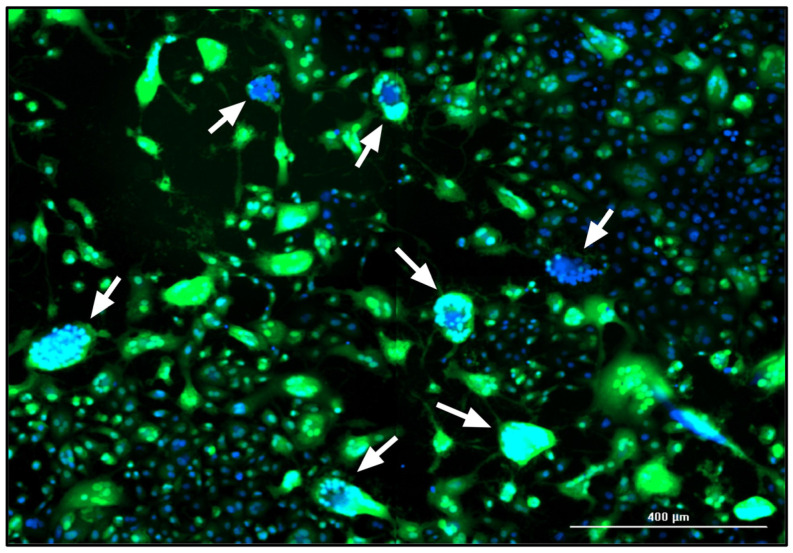
Fluorescence image of a MeV-infected static Vero cell culture in CDM 73835C (MOI = 0.02 TCID_50_ cell^−1^, 5 dpi). The expression of eGFP due to MeV infection is shown in green and cell nuclei are counterstained with DAPI (blue) in this merged image captured using the Cytation 3 (objective magnification: 10×). Arrows point to exemplary syncytia.

In addition to flow cytometry and TCID_50_ assays, the influence of the MOI on static MeV production was also monitored by fluorescence microscopy. The MeV-induced cytopathic effect is illustrated in Figure 4. The fusion of the Vero cells into syncytia was evidenced by the aggregation of nuclei and the large-area GFP fluorescence, which was caused by MeV NSe ld-EGFP infection. In the late stage of the culture process, some areas of the original cell layer were completely lysed, as indicated by the empty areas without stained cell nuclei.

**Figure 5:**
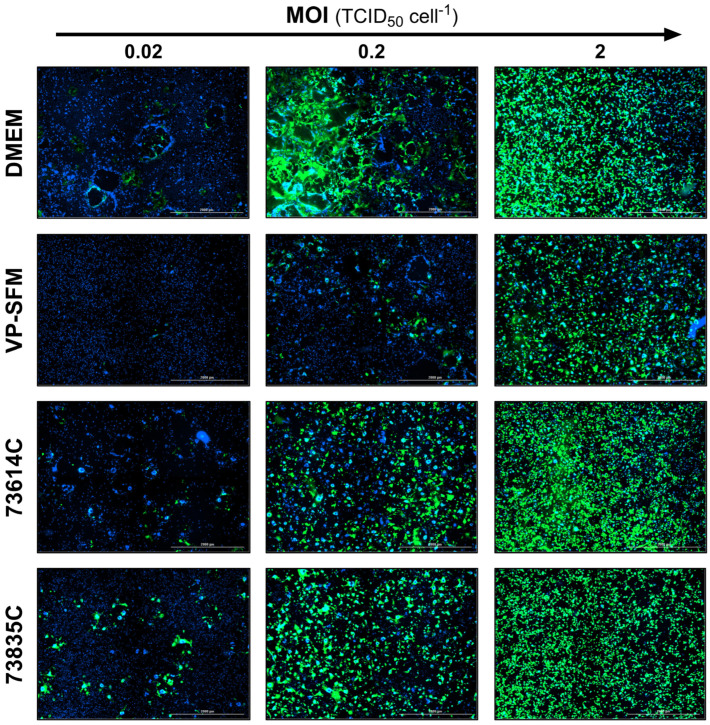
Fluorescence images of MeV-infected static Vero cell cultures 3 dpi in SCM, SFM and CDMs at different MOIs. The expression of eGFP due to MeV infection is shown in green and cell nuclei are counterstained with DAPI (blue) in this merged image captured using the Cytation 3 (objective magnification: 2.5×, four (2 × 2) images per well, stitched).

**Figure 6:**
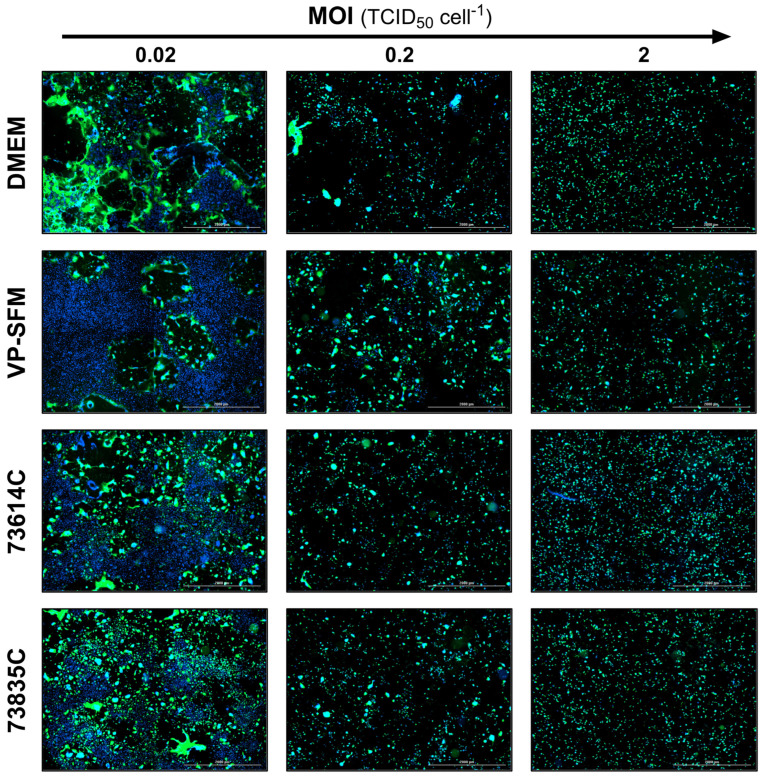
Fluorescence images of MeV-infected static Vero cell cultures 5 dpi in SCM, SFM and CDMs at different MOIs. The expression of eGFP due to MeV infection is shown in green and cell nuclei are counterstained with DAPI (blue) in this merged image captured using the Cytation 3 (objective magnification: 2.5×, four (2 × 2) images per well, stitched).

The levels of GFP expression at the three MOIs tested during the infection course are shown for the different culture media at 3 and 5 dpi (Figure 5 and Figure 6). At 3 dpi, the GFP signal was related to the MOI as anticipated (Figure 5).

At 5 dpi (Figure 6), the green fluorescence and cell number declined for all the media at the highest MOI (2.0 TCID_50_ cell^−1^) compared to 3 dpi, which can be explained by cell lysis due to MeV replication and release. In contrast, the lowest MOI (0.02 TCID_50_ cell^−1^) showed a spread of infection, recognizable by larger GFP^+^ areas. In addition, “infection halos” were visible in the layer in which cells were absent, but which was bordered by green, fluorescent cells. In contrast, some cell-covered areas showed no green fluorescence.

These data suggest that the MeV particles released in static cultivations tend to infect neighboring cells. Furthermore, cell–cell contacts, not only during the formation of syncytia but possibly also through the direct transmission of MeVs at the contact points, could play an important role in the spread of infection in static Vero cell cultures.

### 3.3. Comparison of MeV Production in SCM, SFM and CDMs

To assess the impact of the culture medium on MeV production in more detail, we compared the total cell density and proportion of infected cells as well as the MeV titer in the four different media (Supplementary Materials).

#### 3.3.1. Medium-Dependent Growth Kinetics of Non-Infected and Infected Vero Cell Cultures

As can be seen from the largely similar growth kinetics of the mock-infected control cells (Figure 2), each medium provided sufficient nutrients and sustained a favorable environment for host cell metabolism and proliferation for at least 6 days.

The infected cultures also showed comparable progress in terms of total cell density in all the media at high MOIs (Supplementary Materials). However, the VP-SFM achieved a higher total cell density (~30% higher than the DMEM or the 73835C, and ~100% higher than the 73614C) at an MOI of 0.02. Nevertheless, the number of GFP^+^ (MeV-infected) cells in the VP-SFM during cultivation did not substantially exceed the numbers observed in the other media at any MOI. Taking into account the methodological deviations, such as cell loss during sample preparation, the course of the infected cells at MOIs of 0.2 and 2 was very similar in all four media. At an MOI of 0.02, the number of GFP^+^ cells peaked in all the media at 4–5 dpi, but the peak density in DMEM was 36.0–62.9% lower compared to the other media. As stated in Section 3.2, the number of infected cells was more stable in the DMEM and the CDMs, whereas a sharp decline was immediately observed in the VP-SFM.

#### 3.3.2. Medium-Dependent Infection Kinetics

The influence of the medium on the progress of the infection in the cultures was assessed by estimating the percentage of GFP^+^ cells (Supplementary Materials). The infection spread fastest in the CDM 73614C (50% GFP^+^ cells reached after 1.1, 2.4 and 3.7 dpi at MOIs of 0.02, 0.2 and 2, respectively), closely followed by the CDM 73835C (1.2, 2.6 and 3.9 dpi). In DMEM (10% FBS) and VP-SFM, the infection states were reached slightly later, with the biggest difference (+0.9 days offset) in the VP-SFM with an MOI of 0.02. The slower spread of infection in the VP-SFM explains the prolonged proliferation of the host cells at an MOI of 0.02 compared to the other media.

The initially slower spread of infection in the DMEM compared to the CDMs may reflect the neutralization of viruses by serum proteins, which are added to the DMEM in the form of FBS. An interaction between serum proteins (e.g., complement system or acute phase proteins) and MeVs might prevent the virus from binding to cell-surface receptors and thus inhibit further infection (especially at lower MOIs). However, given that a different MeV stock was applied in each medium, and the TCID_50_ assay has an error of ±0.5 log_10_ [21,22,23], we anticipate some variation in terms of initially infected cells at the supposedly same MOI. Surprisingly, the proportion of GFP^+^ cells revealed the consistent dynamics of infection spreading in the SCM, the SFM and the CDMs, as shown by the similar slopes of the sigmoidal fits (Section 3.2). This suggests that the infection course is to a great extent independent of the medium.

#### 3.3.3. Medium-Dependent MeV Yield

Despite the partly different evolution and maximum number of infected cells, there were only small differences between the media in terms of the maximum infectious MeV titer, even when varying the MOI. The maximum titer was 0.61–1.08 × 10^6^ TCID_50_ mL^−1^ at an MOI of 0.2 and 0.46–2.17 × 10^6^ TCID_50_ mL^−1^ at an MOI of 2. However, the titer range was somewhat wider (0.09–1.08 × 10^6^ TCID_50_ mL^−1^) at an MOI of 0.02. In the VP-SFM, the maximum MeV yield was 0.5–1.0 log_10_ lower than in the DMEM and the CDMs. Therefore, the high density of infected cells in the VP-SFM and the subsequent sharp drop in cell count did not lead to a virus boost. Depending on the culture medium, the maximum MeV yield was observed after different process times. For example, at an MOI of 0.2, the optimum TOH in the CDMs was reached 1–2 days earlier (4 dpi) than in the VP-SFM and DMEM. Interestingly, this correlated with an earlier spread of infection in the CDMs. MeV stability in all the tested media can thus be considered very similar. Although the exact composition of the media is not known, the stability of the MeV titer in the VP-SFM and the CDMs over time may reflect the presence of additives. Ideally, a cell culture medium for the production of MeV should contain additives that protect the virus particles against thermal and mechanical inactivation, and prevent virus aggregation [34,35]. The amino acid composition of the medium also influences MeV synthesis in HeLa cells [36].

Serum is an important source of vitamins, growth factors, hormones, amino acids, adhesion factors and cytokines in cell culture media [9]. The addition of 10% FBS resulted in a 0.5–1.5 log_10_ increase in the titer of the Edmonston MeV strain in the Vero cells compared to the medium without serum [33]. However, the extent to which serum has a positive effect on the MeV yield appears to depend on the culture medium. We observed no major detrimental effects on the MeV yield or process time when using the VP-SFM or Merck CDMs compared to the SCM. Even in a STR, MeV production in VP-SFM at low to moderate shear stress conditions (below 0.25 N m^−2^) resulted in yields similar to the production in the SCM [15]. Furthermore, no significant difference in MeV production was observed in the CDMs Hektor and InVitrus (both Cell Culture Technologies, Switzerland) compared to the VP-SFM [34].

## 4. Conclusions

We found that the combination of MOI and growth medium influences both MeV infection and release. The CDMs were as suitable as the SCM for MeV production, which overcomes all the negative effects of the FBS during production (e.g., batch-to-batch variation and ethical and safety concerns surrounding the use of animal serum). The time course of MeV release was faster in the CDMs, especially in the low and medium MOI range, which was in accordance with a faster spreading of the infection. Nevertheless, further investigation is required to assess the performance of the CDMs in dynamic bioreactors, specifically in flow conditions with higher shear forces, because serum proteins have been shown to protect the shear-sensitive MeV in this environment [15]. We also need to assess how the CDMs affect the stability of the MeV during purification by chromatography [22,23,37] or filtration [23,38,39]. For commercialization, the formulation of the MeV is also an issue in terms of viral stability and the avoidance of aggregation.

The potential for flow cytometry to detect the Vero cells infected with the MeV NSe ld-EGFP would provide a deeper understanding of the production process. The ratio of infected cells can be monitored to determine the most appropriate MOI for an optimal space–time yield of infectious MeV. However, the use of this method to monitor MeV production in STRs may prove challenging, given that adherent Vero cells growing on microcarriers such as Cytodex 1 can be difficult to detach. The aforementioned method would be more applicable to Vero cells adapted for growth in suspension [40,41,42] (Eckhardt et al., data to be published). Fluorescence microscopy offered further insights in the process of MeV spreading and is recommended in carrier-based STR processes to monitor the MeV infection and propagation efficiency during production. Our results confirmed that it is possible to transfer a static MeV production process from a SCM to a SFM or even CDM, resulting in comparable high MeV titers.

## Data Availability

The data presented in this study are available on request from the corresponding author.

## Supplementary Materials

The following supporting information can be downloaded at https://www.mdpi.com/article/10.3390/v16111740/s1, Figure S1: Measles virus production in adherent Vero cells using different culture media (SCM, SFM and CDM) in six-well plates. Following the infection of Vero cells with MeV, the cultivation temperature was reduced from 37 to 32 °C and a complete media exchange was carried out ~1 day later. The concentration of total cells and the ratio of infected cells (%GFP^+^ cells) at different MOIs were determined by flow cytometry, allowing the recalculation of cell densities. Criterion GFP^+^: fluorescence intensity greater than the mock-infected control. Sigmoidal curve fitting of %GFP^+^ cells over the time, forced through points [–0.82;0] and [0;0] (time points of cell seeding and MeV infection). MeV titers were determined using the cell-based 50% endpoint dilution method (TCID50 assay). LOD: limit of detection. The data points correspond to those in Figure 2, but have been presented differently here for a better comparison of the media.
