# Supplementary material for: Production of Oncolytic Measles Virus in Vero Cells: Impact of Culture Medium and Multiplicity of Infection
Source: Viruses. 2024 Nov 6;16(11):1740. doi: 10.3390/v16111740 (PMC11599022; doi:10.3390/v16111740)
Supplement: Supplementary file 1 [file viruses-16-01740-s001.zip › viruses-3242710-supplementary.pdf]

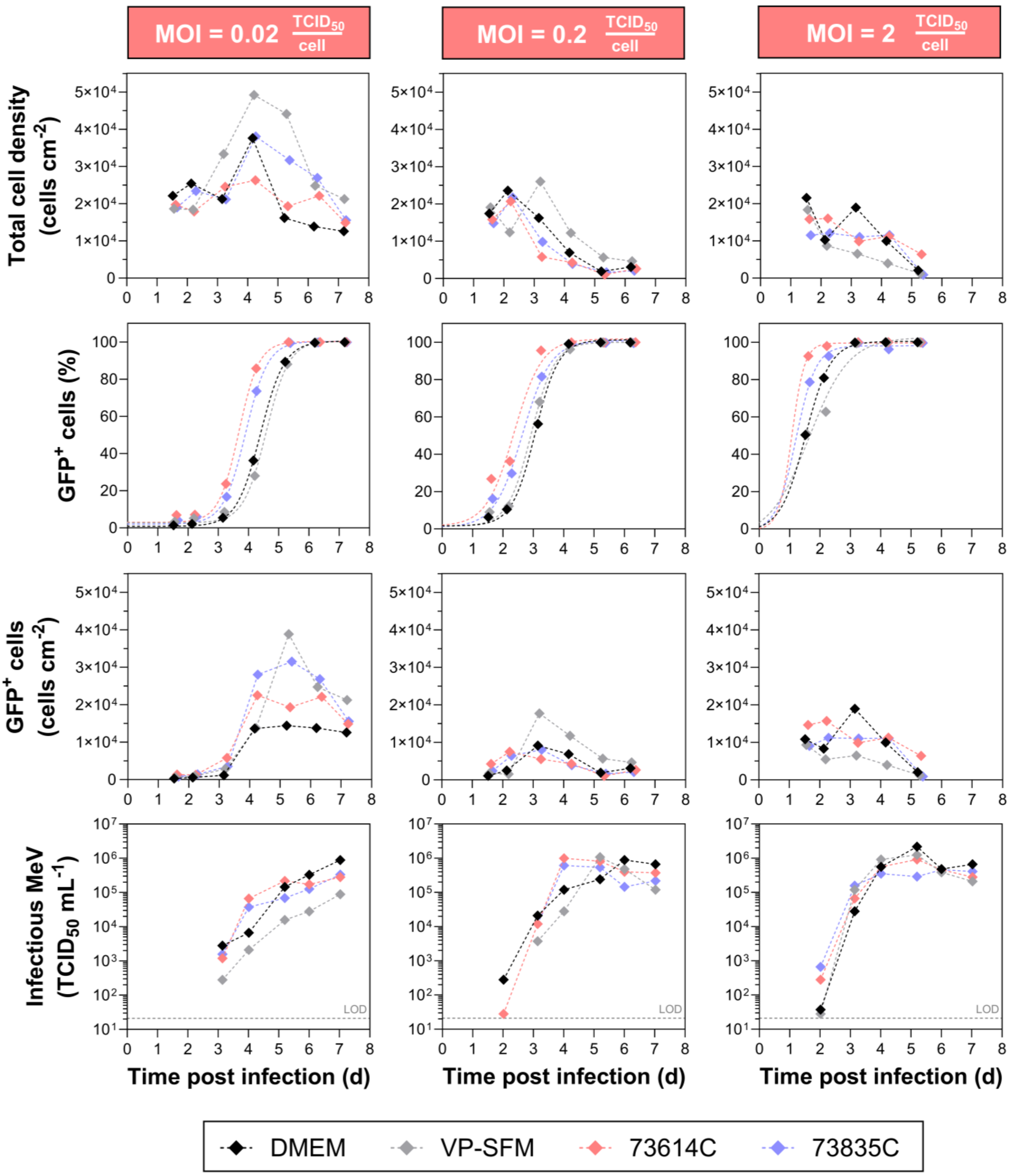

Figure S1 - Measles virus production in adherent Vero cells using different culture media (SCM, SFM and CDM) in six-well plates. Following the infection of Vero cells with MeV, the cultivation temperature was reduced from 37 to 32 °C and a complete media exchange was carried out ~1 day later. The concentration of total cells and the ratio of infected cells (%GFP+ cells) at different MOIs were determined by flow cytometry, allowing the recalculation of cell densities. Criterion GFP+: fluorescence intensity greater than the mock-infected control. Sigmoidal curve fitting of %GFP+ cells over the time, forced through points [–0.82;0] and [0;0] (time points of cell seeding and MeV infection). MeV titers were determined using the cell-based 50% endpoint dilution method ($TCID_{50}$ assay). LOD: limit of detection. The data points correspond to those in Figure 2, but have been presented differently here for a better comparison of the media.
